# Cuttlefish-Bone-Derived Biomaterials in Regenerative Medicine, Dentistry, and Tissue Engineering: A Systematic Review

**DOI:** 10.3390/jfb15080219

**Published:** 2024-08-05

**Authors:** Rihab Adel Al-Rawe, Hasan M. AL-Rammahi, Arief Cahyanto, Azman Ma’amor, Yih Miin Liew, Prema Sukumaran, Wan Nurazreena Wan Hassan

**Affiliations:** 1 Department of Paediatric Dentistry and Orthodontics, Faculty of Dentistry, Universiti Malaya, Kuala Lumpur 50603, Malaysia; rehab_shukur@aliraqia.edu.iq; 2 College of Dentistry, Al-Iraqia University, Baghdad 10011, Iraq; 3 Department of Conservative Dentistry, Faculty of Dentistry, University of Babylon, AL Hillah City 51002, Iraq; dent.hasan.mohsin@uobabylon.edu.iq; 4 Department of Restorative Dentistry, Faculty of Dentistry, Universiti Malaya, Kuala Lumpur 50603, Malaysia; 5 Department of Restorative Dentistry, College of Dentistry, Ajman University, Ajman P.O. Box 346, United Arab Emirates; 6 Department of Chemistry, Faculty of Science, Universiti Malaya, Kuala Lumpur 50603, Malaysia; azman2111@um.edu.my; 7 Department of Biomedical Engineering, Faculty of Engineering, Universiti Malaya, Kuala Lumpur 50603, Malaysia; liewym@um.edu.my; 8 Faculty of Dentistry, Oral & Craniofacial Sciences, King’s College London, London Bridge, London SE1 9RT, UK; prema.sukumaran@kcl.ac.uk

**Keywords:** biomedical, cuttlebone, regenerative medicine, bone regeneration, tissue engineering

## Abstract

Background: Marine ecosystems, covering 70% of Earth’s surface, hold immense biodiversity and potential for biomaterials. Cuttlefish bone (CB) and marine resources have gained attention as eco-friendly biomaterials. Objectives: We aim to comprehensively study biomedical applications of CB-derived materials. By evaluating both in vivo and in vitro investigations, the review seeks to uncover the diverse potential of CB in the biomedical field. Methods: A comprehensive search of electronic databases yielded 51 articles from 2408 studies. These studies encompassed in vivo animal studies and in vitro investigations. Results: In vivo studies employed for bone repair, dorsal subcutaneous defects, thermal wound healing, muscle injections, and avian blood testing. In vitro studies focused on HAp synthesis, scaffold development, dental material enhancement, and antimicrobial properties. Risk of bias assessments revealed varying degrees of methodological quality in both animal and in vitro studies, underscoring the need for standardised reporting and rigorous study design in future research. Conclusions: This review fills a gap in the literature by providing a comprehensive overview of the applications of CB-derived materials in the biomedical field. Additionally, it offers valuable insights for researchers, clinicians, and policymakers interested in sustainable and effective biomaterials for diverse medical purposes, advancing the fields of regenerative medicine and dentistry.

## 1. Introduction

The diversity of marine life is enormous, with an estimated 200,000 species already known and countless yet-undiscovered wonders occupying more than 70% of our planet’s surface [1]. This aqueous expanse lies a treasure trove of materials, particularly calcium carbonate polymorphs that are intertwined with various marine structures. Marine-derived ceramic materials offer several benefits, including non-toxicity and higher availability, sparking interest among specialists in various biomedical applications [2]. These marine-derived biomaterials possess distinctive attributes, including low electrical and thermal conductivity, minimal toxicity, exceptional biocompatibility, biodegradability, and remarkable resistance to compression. Their surface chemistry and unique bone-bonding properties position them as ideal candidates for applications in bone substitution engineering and medicine [2]. Marine sources’ distinctive shape and bioactive components enable their usage in tissue engineering and drug delivery systems, allowing biomimetics and intelligent biomaterials tailored for personalised medicine [3].

Utilising marine byproducts for biomaterials reduces reliance on synthetic alternatives, lowers costs, and mitigates environmental harm, promoting ecosystem health. This approach also prevents environmental contamination by reducing biomass waste accumulation [4]. Additionally, nanoparticles like silver, titanium, and gold derived from marine sources have various applications in pharmaceuticals, environmental management, cosmetics, and drug delivery [5]. Moreover, the synthesis of these materials often aligns with green synthesis approaches, which prioritise environmentally friendly and sustainable methods [6]. By adopting green synthesis techniques, the development of marine-derived biomaterials can be both innovative and ecologically responsible.

Recent advances in the development of green and marine biomaterials underscore the significant scientific interest in utilising marine-derived matrices and mineral powders for biomedical applications. For instance, Gandolfi et al. [7] developed innovative green hydrogels composed of sodium mannuronate/guluronate, gelatin, and biointeractive calcium silicates/dicalcium phosphate dihydrate specifically designed for the regeneration of oral bone defects through their bioactive properties. Similarly, Yan et al. [8] created an injectable alginate/hydroxyapatite gel scaffold combined with gelatin microspheres, demonstrating its effectiveness for bone tissue engineering applications. This composite material leverages the osteoconductive properties of hydroxyapatite and the biocompatibility of alginate to enhance osteoblast activity and in turn bone tissue regeneration. These examples illustrate the growing trend and promising future of marine-derived biomaterials in advancing regenerative medicine and tissue engineering.

Delving into the marine sources, we encounter a rich mosaic of organisms like coral, mussels, oyster seashells, crab shells, and sponges [9,10,11,12,13,14,15]. Among these, corals, marine invertebrates adorned with calcium carbonate (CaCO_3_) skeletons have earned notable attention since the 1970s. Their commercial production of HAp for bone graft materials, underpinned by porous structures and intricate pore interconnections, showcases excellent mechanical bonding and biocompatibility, signifying promise in bone substitution engineering and medicine [16]. The conversion of coral to coralline HAp still keeps its unique microstructure. This biomaterial has osteoconductivity, bioactivity, and biocompatibility, allowing for direct bone-to-biomaterial coupling and gradual host-bone replacement following implantation [17]. Nevertheless, this technology’s substantial drawback lies in its impact on coral reefs, vital bastions for marine life and biodiversity conservation [18].

Cuttlefish bone (*Sepia officinalis*), alternatively known as cuttlebone (CB), has emerged as a compelling source of bioceramics characterised by chemistry and crystallography akin to corals [19]. A comprehensive exploration of marine habitats has unveiled CB’s vast potential, particularly in biomedical applications. CB’s significance lies in its composition of natural aragonite, a crystallised form of CaCO_3_ readily convertible to HAp using simple methods [20]. 

This marine resource boasts global accessibility, affordability, interconnected pore structures, and adaptable sizing properties, rendering it well suited for supporting diverse physiological processes. Furthermore, cuttlefish bone (CB) exhibits exceptional machinability, facilitating rapid customisation to meet specific requirements. It demonstrates robust in vitro bioactivity and boasts high biocompatibility, as corroborated by osteoblast tests. Additionally, AB-type carbonated hydroxyapatite, which closely mirrors the composition of human bones, can be obtained from CB after specific treatments [21]. However, its application in bone tissue engineering faces limitations due to CB’s inherent brittleness and low strength. These challenges can be mitigated by incorporating varying concentrations of polymers into the scaffold [22].

CB’s captivating structure further piques scientific curiosity. Comprising a fragile ventral (interior) shell and a robust dorsal shield, CB features an interconnected chamber-like architecture with high porosity, contributing to the cuttlefish’s buoyancy at various depths during underwater sojourns [15,23]. These microchambers within CB exhibit size variations linked to their specific locations within the structure. Horizontal striations adorn the arches, giving them a parallel, wave-like appearance. These arches account for CB’s high porosity (approximately 93 percent porosity), encompassing pores ranging from 200 to 600 µm, which regulate CB’s water content [24].

CB, a naturally occurring substance, showcases multifaceted attributes, including high porosity, remarkable flexural stiffness, and compressive strength, exemplifying nature’s prowess in designing optimised cellular structures [25]. Its intricately organised interior shell, composed of aragonite polymorph fused with a small amount (3 to 4.5 wt%) of organic matter, primarily β-chitin and protein, enhances CB’s utility in templating inorganic growth [26]. The crux of regenerative medicine lies in restoring healthy biological processes, spanning from cellular to tissue-level regeneration, culminating in repairing or replacing damaged or diseased cells, tissues, and organs. Notably, HAp, alpha-tricalcium phosphate (α-TCP), and beta-tricalcium phosphate (β-TCP) play pivotal roles in bone grafting for bone tissue regeneration and repair. Crafting 3D HAp-based scaffolds was a premier technique for producing bone graft materials [27]. The versatility of CB-derived HAp is a beacon in regenerative medicine, enabling the creation of composite materials that mimic the natural properties of bone. These composite materials have yielded superior bone growth, repair, and regeneration [1].

The superior cellular structure, biological nature, and mechanical behaviour of CB, along with its CaCO_3_ (aragonite) composition, make it highly suitable for HAp and scaffold synthesis. Unlike nacre and coral, which collapse after hydrothermal transformation due to high temperature and pressure, CB maintains its stability [28]. Additionally, the presence of chitin, which is biocompatible, biodegradable, hemostatic, and antimicrobial, enhances its suitability for biomedical applications. The essential properties of cuttlefish-based biocomposites, such as high porosity, optimal modulus of elasticity, biocompatibility, strength, and non-toxicity, further contribute to its advantages over other marine-based bio-ceramics like corals, sea sponges, sea urchins, crab shells, and oyster shells, particularly in bone tissue engineering and repairing bone defects with its excellent mechanical strength [15,29]. Table 1 summarises the key differences and similarities between CB and other marine biomaterials.

Remarkably, despite the promise of CB in biomedical fields, a systematic review to comprehensively explore its applications in these disciplines remains conspicuously absent. This systematic review embarks on a journey to illuminate the landscape of in vitro and in vivo studies investigating the biological applications of CB in tissue engineering, regenerative medicine, and dentistry. By shedding light on this uncharted territory, this paper aims to comprehensively explore the published literature about the potential biological application of cuttlefish bone-derived materials, expanding the knowledge about these promising biomaterials and opening new horizons for future medical and dental research endeavours.

## 2. Materials and Methods

### 2.1. Protocol and Registration

This systematic review adheres to the Preferred Reporting Items for Systematic Reviews and Meta-Analyses (PRISMA) guidelines and was registered with the Open Science Framework Database (https://osf.io) on 15 January 2023 and possesses a registration DOI of https://doi.org/10.17605/OSF.IO/2VA8W.

### 2.2. Research Question and Search Strategy

The research question guiding this review is “Is the use of cuttlefish bone biomaterials effective in biomedical applications?”. To compile relevant studies, an extensive search strategy was implemented across three prominent electronic databases: Scopus, PubMed, and Web of Science. The search query employed a comprehensive array of keywords with interchangeable usage, alternate spellings, synonymous expressions, and MeSH terms. The search strategy was integrated with the use of Boolean operators (i.e., “OR” and “AND”). The keywords, which closely aligned with the objective of our study, included the following: (“cuttlebone” OR “cuttlebones” OR “sepia officinalis” OR “cuttlebone powder” OR “cuttlebone hydroxyapatite” OR “cuttlebone hydroxyapatites” OR “calcium carbonate” OR “aragonite” OR “cuttlebone aragonite” OR “CaCO_3_”) AND (“tooth bioengineering” OR “bone tissue engineering” OR “hard tissue treatment” OR “bone graft” OR “scaffold” OR” scaffolds” OR “bone scaffold” OR “bone tissue engineering scaffolds” OR “natural biomaterial” OR “marine biomaterial” OR “marine biomaterials” OR “bio-ceramic materials” OR “bioceramic materials” OR “bio-ceramic scaffolds” OR “bioceramic scaffolds” OR “biomedical application” OR “biomedical uses” OR “regenerative medicine” OR “regenerative dentistry”). 

This process commenced on 16 January 2023 and was updated on 17 March 2024, encompassing studies published from 2000 to 2024. While research on this topic dates back to the 1970s, significant advancements in HAp scaffold preparation began with Rocha, Lemos [37]. Therefore, this date range captures the most crucial period for HAp scaffold synthesis using CB. 

### 2.3. Data Processing

After conducting a database search to extrapolate the findings, two reviewers, R.A.A. and H.M.A., independently assessed the studies based on selection criteria that fit the purpose of the review, The selected studies were downloaded in the EndNote software (Version 21 (Bld 17096), Clarivate, London, UK). Four rounds of screening and filtering were performed to evaluate the selection process. First, duplicates were removed. Second, the search results were appraised for relevance based on their titles. Third, unrelated articles were removed after screening the abstracts. Finally, the full texts were read and analysed.

### 2.4. Inclusion and Exclusion Criteria

Predefined inclusion and exclusion criteria guided the final selection of studies for in-depth examination. As for the type of publication, only research studies were included. All conference papers, abstracts, pilot studies, reviews, communications, letters to the editor, and editorials were excluded. Studies published in languages other than English were also excluded to ensure relevance and consistency. As for the study relevance, only studies (in vivo and in vitro) related to biomedical applications of CB were included. Studies dealing with the fabrication or synthesis of a wide variety of materials, material compositions, and applications other than biological ones were excluded. Considering the materials of interest, studies on tricalcium phosphate, eggshells, chitosan, bioglass, marine sources other than CB, and industrial applications of CB were also excluded. The eligibility criteria were then applied, excluding studies that focused solely on physical, mechanical, and chemical properties without any medical application, as well as studies that dealt only with morphology and tribological behaviour. Two independent reviewers (R.A.A. and H.M.A.) comprehensively assessed the full text of the selected studies based on strict eligibility criteria. Discrepancies in study inclusion or exclusion were resolved through consensus among the reviewers. The inter-reviewer agreement was evaluated using Cohen’s kappa coefficient [38]. 

**Figure 1:**
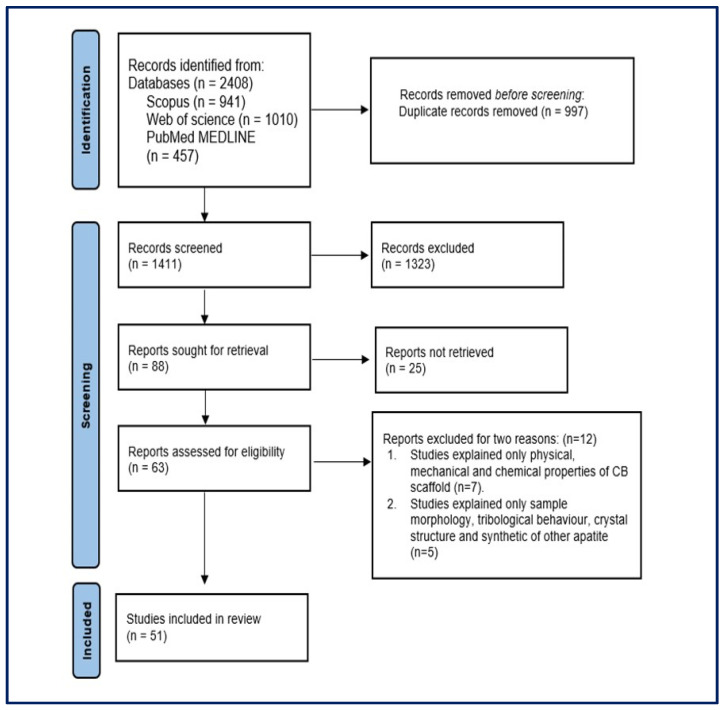
Preferred Reporting Items for Systematic Reviews and Meta-Analyses (PRISMA) flowchart of the included studies.

A comprehensive depiction of the study search and selection process is presented in Figure 1, following the PRISMA guidelines. The two reviewers verified the accuracy and alignment of the extracted information for the selected studies. The data were extracted from each of the selected studies and then organised, summarised, and tabulated, including details such as the author(s), year of publication, the aim of the study, methodology (preparation of CB, characterisation tests of the materials, and preparation of the animal), findings, and study limitations.

### 2.5. Risk of Bias (ROB) Assessment

The assessment of the ROB entailed a systematic categorisation of the final dataset based on study design, resulting in two principal groups: in vivo (animal studies) and in vitro investigations. These assessments were independently conducted by two reviewers, R.A.A. and H.M.A.

For animal studies, SYRCLE’s ROB tool, which is rooted in the Cochrane ROB tool, was adapted to account for bias unique to animal intervention studies [39]. This tool encompassed multiple domains to evaluate various facets of bias, including selection bias (with components like sequence generation, baseline characteristics, and allocation concealment), performance bias (encompassing random housing and blinding procedures), detection bias (evaluating randomisation and blinding in outcome assessment), attrition bias (assessing incomplete outcome data), reporting bias (scrutinising selective outcome reporting), and other potential sources of bias. Signal questions were integral to this evaluation, with responses categorised as “Yes” indicating a low risk of bias, “No” signifying a high risk of bias, and “Unclear” indicating insufficient information to accurately assess bias risk.

Conversely, in vitro studies underwent evaluation using the QUIN (Quality Assessment Tool for In Vitro Studies) ROB tool. This standardised tool for assessing bias in in vitro studies incorporated 12 domains to evaluate study quality [40]. These domains encompassed clarity of aims/objectives, detailed sample size calculation, elucidation of sampling techniques, descriptions of the comparison group, operator specifications, randomisation, outcome measurement methods, details on outcome assessors, blinding, statistical analysis, and presentation of results. Scoring entailed assigning 2, 1, or 0 based on whether a criterion was well specified, inadequately specified, or not applicable, respectively. The final score for each study was calculated using a specific formula:

Final score = (Total score × 100)/(2 × Number of applicable criteria)

The calculated scores were interpreted as follows: a score of >70% = low ROB, 50–70% = medium ROB, and <50% = high ROB. These comprehensive assessments aimed to enhance the credibility and validity of this systematic review, ensuring a robust foundation for data interpretation and synthesis.

## 3. Results

### 3.1. Study Selection

The comprehensive search query resulted in a total of 2408 research publications across three prominent databases: 941 from Scopus, 457 from PubMed, and 1010 from Web of Science. After eliminating duplicate studies, 1411 studies remained and were subjected to further scrutiny. Of the initial studies, 1323 were excluded based on irrelevant titles, leaving 88 studies for more detailed examination. After a thorough review of abstracts, 25 non-relevant studies were excluded from consideration. The remaining 63 studies underwent a comprehensive evaluation by reviewing their full texts based on predefined eligibility criteria. A total of 12 studies were excluded for two main reasons as shown in Figure 1. Of those, seven studies focused only on the physical, mechanical, and chemical properties of the CB scaffold without mentioning any medical application. The other five studies addressed only sample morphology, tribological behaviour, crystal structure, and the synthetic nature of apatites. Following the application of these eligibility criteria to all studies, the final dataset for this systematic review encompassed 51 studies. The inter-reviewer agreement for inclusion and exclusion studies based on Cohen’s kappa coefficient demonstrated substantial agreement (kappa = 0.74).

### 3.2. Literature Taxonomy

**Figure 2:**
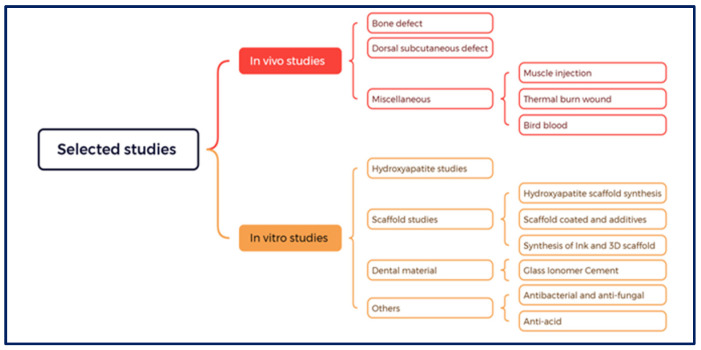
Taxonomy of the included studies.

To facilitate future research and address various associated issues and scientific gaps, a taxonomy was developed based on the utilisation of CB in biomedical applications as shown in Figure 2. This taxonomy classified studies into in vivo and in vitro categories.

#### 3.2.1. In Vivo Category

In vivo studies involving animal experiments, encompassing rabbits, rats, or birds, accounted for 16 studies (16/51). These studies were further categorised into three main groups. The first group of studies focused on animal bone structure, defect, or fracture (11 studies) at diverse anatomical sites including the calvaria, tibia, and sinus. The second group included studies that addressed dorsal subcutaneous defects (two studies). The miscellaneous group (third one) was further subdivided into three subgroups that examined thermal wound healing, muscle injection for tested substances, and bird blood testing (one study each). Detailed characteristics of these in vivo studies are presented in Table 2.

#### 3.2.2. In Vitro Category

This category was subdivided into four main groups: the HAp group, scaffold group, dental materials group, and others.

##### HAp Group

The studies in this group (7/51 studies) focused on synthesising and characterising HAp as a potential biomaterial for bone tissue engineering (BTE). These studies explored the properties and suitability of HAp for biomedical applications. The study characteristics of this group are presented in Table 3.

##### Scaffold Group

The main research subject in this group of studies (21/51 studies) was the synthesis, characterisation, and biomedical application of scaffolds. Accordingly, this group of studies was further subdivided into three subgroups. The first subgroup included five studies that mainly dealt with the pure HAp scaffold synthesis, revealing its importance for tissue engineering or direct clinical use. The second subgroup had eight studies that dealt with scaffold coating, which improves the materials, and mechanical properties. The synthesis of ink and 3D scaffolds was the main area of interest in the third subgroup (eight studies). Characteristics of studies belonging to this group are presented in Table 4.

##### Dental Material Group

Three out of fifty-one studies were assigned to this group, which mostly focused on glass ionomer cement (GIC). These studies specifically addressed the incorporation of CB-derived HAp powder into GIC, assessing the potential enhancement in its physical properties. Table 5 shows the characteristics of this study group.

##### Others

This group included four out of fifty-one studies. Three studies explored the antibacterial and antifungal effects, discussing natural sources of safe antimicrobial agents. One study focused on synthesising antiacid substances from CB raw material. The study characteristics are explained in Table 6.

### 3.3. Risk of Bias Assessment

**Figure 3:**
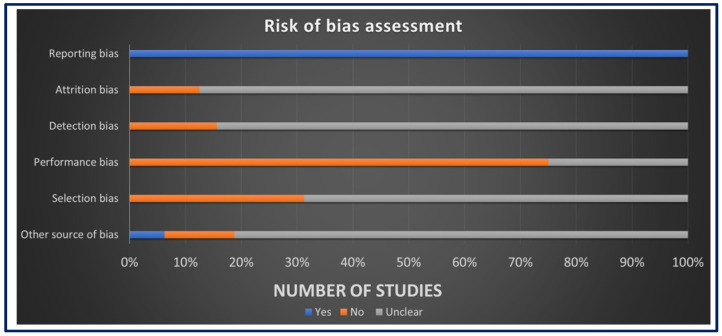
The risk of bias assessment using SYRCLE’s tool.

**Figure 4:**
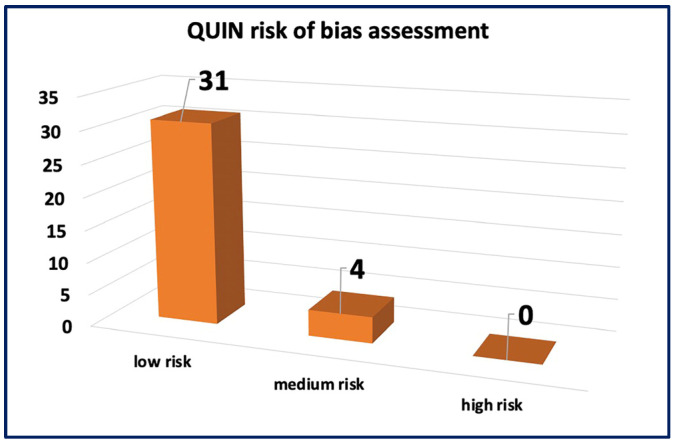
The risk of bias assessment using the QUIN tool.

The assessment of ROB for in vivo studies was conducted using SYRCLE’s ROB tool as seen in Figure 3. The results revealed a low ROB in the reporting bias domain, attributed to the comprehensive reporting of primary and secondary outcomes and well-clarified methods and results sections in all studies. However, for other domains, such as allocation concealment, random housing, blinding, and random outcome assessment, more than 50% of the studies displayed an unclear ROB, mainly due to insufficient data and less likely due to insufficient details on housing conditions and the timing of outcome assessment. The assessment of ROB for in vitro studies was performed using the QUIN ROB tool as seen in Figure 4. Among the 35 studies assessed, 31 studies exhibited a low ROB, with final scores exceeding 70%. Four studies were classified as having a medium ROB with final scores exceeding 60%. These findings indicate that most studies provided clear aims, objectives, comparison groups, detailed methodologies, outcome assessments, statistical analyses, and results. However, some domains did not apply to these studies. These comprehensive assessments collectively contribute to the credibility and reliability of this systematic review, establishing a robust foundation for the interpretation and synthesis of data. 

## 4. Discussion

CB has attracted a tremendous deal of interest from scientists who are working to understand its special characteristics and consider its potential applications. Numerous studies have delved into CB’s chemical and mechanical structures, revealing versatile attributes that can be harnessed for biological and industrial endeavours [25,28,87,88,89,90]. CB stands as an economical, abundantly available, and remarkably bioactive natural material, poised for transformation into HAp and bone-mimetic scaffolds. In the fields of tissue engineering and regenerative medicine, the utility of CB scaffolds has been increasingly investigated [91]. In pursuit of the ideal bone substitute—one that avoids immune responses while remaining bioactive, osteoconductive, osteoinductive, biodegradable, sterilisable, accessible, and cost-effective—CB emerges as a promising alternative to autogenous bone grafts [92]. To shed light on this prospect, our discussion segment will emphasise in vivo and in vitro studies that have examined the application of CB in biomedical and regenerative contexts. 

### 4.1. Application of CB in an In Vivo Setting

The conversion of CB-derived materials into augmenting biomaterials for bone defects or as potential autogenous bone substitutes has garnered substantial research attention. It has been conclusively established that CB material holds promise as a bone graft material, BTE scaffold, bone filler, and regenerating bone. CB-based derivatives, which were mostly synthesised based on hydrothermal techniques, have been distinguished by their excellence in serving as bone substitutes either solely or in combination with other biological materials [43,44].

It has been reported that the most optimal osteogenic differentiation has been observed with CB-derived scaffolds in aerogel form, which fosters cell adhesion, proliferation, alkaline phosphatase (ALP) activity, targeted gene expression, and mineralisation. Surface roughness modifications have been shown to enhance cell adhesion, osteoblast proliferation, and differentiation [42]. Biocompatibility assessments of CB typically involve scrutinising its potential cytotoxicity impact on MG-63 cells (osteoblast-like human osteosarcoma cells) [52], as well as evaluating the adhesion and growth of human marrow mesenchymal stem cells (hMSCs) on the scaffold [54]. The extraordinary topographical structure of these substances enables the synthesis of novel periosteum substitutes that demonstrate remarkable osteogenic and angiogenic properties [41]

The porous nature of CB, with its capacity for blood circulation and support for bone cell adhesion, proliferation, and nutrient solution microcirculation, has been lauded for its ability to boost neovascularisation and bone tissue formation [45,52,54]. Moreover, the high surface area for bone–material interaction in CB-based HAp augments the biomechanical environment, creating favourable conditions [46]. CB exhibits remarkable biocompatibility, osteoconductivity, and bioactivity, with no discernible rejection, infection, or cytotoxic effects on bone healing. Also, it reduces free radicals in soft tissues and enhances bone healing without triggering oxidative stress [45,53].

Histologically, CB-based materials have accelerated callus formation and increased osteoblast production, whether used as bone graft materials or as subcutaneous implants, thus hastening the bone repair process. [47]. The initiation of bone healing involves fibrous tissue formation followed by vascularisation and osteochondral union. CB-based materials have demonstrated exceptional effectiveness in promoting bone union, achieving 100% bone union in radiographic assessments [53]. Moreover, the encapsulation of the grafting material has led to enhanced inflammatory cell infiltration, which further enhances osteoconduction [18].

CB-based HAp graft materials have shown osteoconductivity and biocompatibility through observations of inflammatory reactions around the implanted material, the proliferation of fibroblasts and connective tissue, and the eventual encapsulation of the implant [51]. Additionally, cell infiltration and proliferation have shown a non-toxic effect of CB material when implanted in bone or muscle, comparable to that of synthetic bone grafts [49,50]. CB has also demonstrated substantial influence on healing skin burns and wounds, enhancing re-epithelialisation, granulation tissue formation, hemostasis, and inflammation inhibition, attributed to chitin polysaccharide action [55]. Of particular interest is the sulphated chitosan (SP-LMWSC) extracted from CB, which has exhibited both in vitro anticoagulant and antiviral properties, indicating its potential utility in various medical applications [48]. CB exhibits a multitude of exceptional properties, including biocompatibility, osteoconductivity, cell adhesion promotion, non-toxicity, osteoblast proliferation, and differentiation. Collectively, these findings robustly advocate for the potential of CB in BTE and augmentation. However, the results should be interpreted cautiously, considering the outcomes of the ROB assessments.

### 4.2. In Vitro Category

#### 4.2.1. Biomedical Application of HAp

HAp [Ca_10_(PO_4_)_6_(OH)_2_] has long been explored as a potential implant material due to its structural resemblance to the mineral component of bone and teeth. The investigation into HAp ceramics derived from natural materials has yielded innovative biomedical applications. HAp’s porous morphology is conducive to vascularisation, bone cell invasion, and angiogenesis. It exhibits rapid absorption by living cells, surpassing stoichiometric HAp, and accelerating bone regeneration [93,94,95].

Given the easy transformation of CB’s aragonite into HAp, it has been used as a biomaterial in BTE and other biomedical applications. Key features, such as the interconnected channelled structure, which facilitates cell growth, nutrient passage, and waste elimination, enhance its biological excellence [32,61]. The biological activity of nanocrystalline HAp derived from CB has been attributed to the presence of different ions such as the strontium ion (Sr^2+^) and magnesium ion (Mg^2+^) [57,60].

To enhance the mechanical strength of HAp, researchers have introduced biological macromolecules like silk fibroin, forming injectable hydrogels. This approach has improved cell proliferation rates and cell-to-cell communication among osteoclasts and osteoblasts. Additionally, CB-based HAp nanocomposites have demonstrated antibacterial activity against microorganisms such as *S. aureus* [59]. It is worth noting that adding carbon and silver nanoparticles has also been practised to significantly increase the mechanical and biological properties, enhance the proliferation rate of MG63 cells, and provide higher antibacterial action against microorganisms (e.g., *E. coli* and *S. aureus*) [58]. The hydrogel derived from CB-based HAp and its non-toxic features has shown exceptional qualities for use in medical substances [56].

#### 4.2.2. Biomedical Application of Scaffold

Scaffolds represent highly specialised biomaterials suitable for tissue engineering and therapeutic use [37]. When analysing marine-derived biomaterials, focusing on bioactivity and biointeractivity properties is crucial. In bone tissue engineering, osteoinductive scaffolds promote bone growth and integration. An ideal pore size for scaffolds in BTE is over 100 micrometres, supporting cell infiltration, vascularisation, nutrient transport, cell adhesion, and growth. These materials possess porous matrices with interconnected pores that facilitate nutrient and oxygen transfer and support cell adhesion, proliferation, and differentiation. Mechanical properties such as stiffness, strength, and toughness are vital for tissue replacement [96]. Additionally, it is crucial to synchronise the scaffold’s degradation rate with the rate of new tissue formation. This ensures the scaffold provides temporary support, is gradually replaced by natural bone, and enhances angiogenesis and osteogenesis for bone tissue engineering [62,70,71]. 

Inspired by the clinical need for simple custom shapes, biomimetic scaffolds have been successfully employed as clinical implants [21,37,79]. Other compositions, such as porous biphasic calcium phosphate (BCP) scaffolds, have shown promise for osteointegration and osteoinduction [20]. CB has emerged as a favourable substrate for cell development and the growth of hMSCs. Its lamellar matrix allows for better cellular penetration than the dorsal shield and it can serve as both a bone replacement and a barrier against fibroblast infiltration near bone defects [78]. To enhance mechanical properties and maintain microstructure, scaffolds are coated with biodegradable polymers [97]. 

Polymers such as polycaprolactone (PCL) and polyvinyl alcohol, poly DL-lactide, a polyester amide (PEA), polyester urea, polylactic acid, and sol-gel improve the mechanical (e.g., hardness, compressive strength, and elastic modulus) and biological (e.g., cell adhesion, proliferation, and differentiation) features of the HAp scaffold for BTE [21,37,79]. On the other hand, unprocessed CB with collagen has been used to synthesise CB-HAp-collagen scaffolds, which can also be beneficial for BTE as it is bioabsorbable and has low immunogenicity [73]. HAp with whitlockite and Mg^2+^, used as porous Mg-substituted calcium phosphate scaffolds, has shown improvements in mechanical and biological properties and can thus be used as a candidate in BTE [67].

Scholars have attempted to create three-dimensional (3D) scaffolds similar to cancellous bone structures for multifunctional purposes. For instance, researchers designed 3D cellulose-based scaffolds [70,72] and regenerating cellulose RC/CB scaffolds [68] with simulated body fluid (SBF) coatings to enhance cell adhesion, promote osteoconduction in BTE, and increase calcium phosphate deposition. This was facilitated by the ability of β-chitin to bind proteins and trace elements that are crucial for mineralisation. Moreover, sophisticated 3D scaffolds with layer-by-layer deposition that were designed with BioCAD software with crosslinking steps were mechanically efficient and had good energy absorption and low weight, and, as a result, could be used in a wide range of medical applications [66]. Nano-BCP 3D printing powder derived from CB was mixed with glass–ceramic powder and different pore geometries were created with sufficient porosity. This type of scaffold showed quick adherence with hMSCs, which enter the pores and colonise the porous structure [65].

To fully control and create sufficient scaffold porosity, powder 3D printing was confirmed to be an appropriate approach for BTE with good reproducibility. Such scaffolds have been prepared from nano-BCP powder derived from CB mixed with glass–ceramic powder and different pore geometries. [64]. Moreover, paste-like 3D printing inks [63] are made from an extracellular matrix based on CB and from biomineral composite scaffolds of five materials (eggshell, pearl, turtle shell, degelatinated deer antler, and CB) with appropriate porosity [62]. These scaffolds have good biocompatibility and can encourage bone regrowth and stimulate biomineralisation. In a nutshell, the superior properties of the 3D-CB scaffold motivate researchers to apply it as a promising technology in biomedical and regenerative medicine. 

#### 4.2.3. Incorporation of CB-HAp into Dental Materials

Glass ionomer cement (GIC) is one of the restorative materials widely used in dentistry. While its advantages include easy bonding to dental tissue, biocompatibility, ease of handling, and fluoride release, its mechanical properties are relatively low [98]. Consequently, the incorporation of CB-HAp into GIC has improved its mechanical properties. Noteworthy, the incorporation of CB-microparticles was preferable, as it is easier to be mixed with resin [99], compared to nanoparticles, which have rough surfaces (cauliflower-like morphology), extending the GIC’s setting time [100].

Bilić-Prcić et al. [82] showed that compressive strength, flexural strength (FS), and diametral tensile strength of chemically set Fuji IX groups were not improved when manually mixed with micro-HAp (<180 µm) at varied wt% concentrations (2, 5, and 10 wt%). However, the mechanical properties of light-cured Fuji II groups showed improvement, particularly with 10 wt% HAp, which yielded the best results in terms of FS. Additionally, the surface roughness and microhardness were not improved by adding HAp-CB to commercial GICs (Fuji IX and Fuji II). This was not the case for the Fuji II, which had been mixed with a 10 wt% HAp group, and that was attributed to the size of the particles and air inclusion due to manual mixing. However, adding HAp-CB significantly improved the fluoride release property of GIC [81]. 

Ivanišević, Rajić et al. [80] incorporated a mixture of titanium dioxide (TiO_2_) nanoparticles and CB-HAp microparticles into conventional GIC. TiO_2_ at the nanoscale has antibacterial properties, good chemical stability, and high biocompatibility and potentially reinforces GIC. Although the CB-HAp microparticles enhanced GIC fluoride release, there were no improvements in compressive strength, breaking strength, or compressive modulus due to an inadequate powder-to-liquid ratio that left many particles unreacted [80]. 

#### 4.2.4. Incorporation of CB-HAp into Antimicrobial, Antifungal, and Antiacid Agents

Antibacterial activity is a crucial feature in preventing implant failure. HAp may induce rapid bacterial biofilm growth due to bioactive calcium and phosphate that provide nutrients for bacterial colony growth [101]. However, nano-HAp derived from CB has exhibited potent antibacterial activity against Gram-positive *Bacillus subtilis*, surpassing its effectiveness against Gram-negative *E. coli* [84,86]. The mechanism involves the penetration of nanoparticles into the bacterial cell membranes, increasing their osmotic potential and leading to irreversible cellular damage. Because CaCO_3_ and polysaccharides in CB’s chitosan can cause bacterial death by releasing cellular contents, powdered CB has shown effective antibacterial and antifungal properties against *Klebsiella oxytoca* and *Aspergillus flavus*, respectively. However, in as study, no antibacterial action was reported against *Staphylococcus aureus* as it could not dissolve the *S. aureus* peptidoglycan cell wall [83].

Only one study presented the CB as a natural antiacid tablet, overcoming the side effects of synthetic chemical drugs. The results demonstrated that CB outperformed commercially available antacid tablets such as CaCO_3_ and Al-Mg tablets. Excellent antacid properties were reported by using calcined CB, which is characterised by high purity, the absence of organic components, and appropriate physicochemical qualities [85]. CB can be considered a promising material for synthesising affordable, antibacterial, anti-fungal, and antiacid agents.

This systematic literature review has included studies that dealt with different medical applications, whether in vitro or in vivo. However, it has potential limitations as it has included only three databases with no grey literature conducted and is limited to English studies. Additionally, no meta-analysis was performed due to the lack of consistent numerical values from the included studies.

### 4.3. Limitations and Challenges of Clinical Translation of CB

The current research on CB biomaterials primarily relies on preclinical studies involving animal models, which present their own set of limitations. As noted in the tables, these limitations include a lack of mechanical and characterisation tests, the application of materials for short periods, and insufficient comprehensive mechanical, biological, or bioactive tests in some in vitro experiments. To facilitate the translation of these materials to clinical trials and human studies, it is crucial to examine their long-term immunogenicity to evaluate potential adverse reactions and immune responses over extended periods. Assessing their efficacy and safety in practical medical settings is also essential. Addressing these challenges is vital for overcoming the current evidence base limitations and ensuring the successful clinical application of CB biomaterials.

Translating findings into clinical practice presents significant challenges. Regulatory approval requires extensive preclinical and clinical data to meet safety and efficacy standards. Clinical validation demands robust trials with adequate sample sizes and follow-up durations to establish reliability and effectiveness. The long-term monitoring of patients with CB implants is crucial for assessing their safety, efficacy, and potential complications over extended periods. Additionally, understanding CB’s degradation kinetics and biostability in physiological conditions is essential for predicting its durability and performance in vivo. Despite the promising mechanical properties of CB biomaterials, such as their high porosity and stiffness, optimising these characteristics for specific biomedical applications, like load-bearing implants or tissue engineering scaffolds, remains challenging. Standardising processing methods is essential to ensuring reproducibility and consistency of results across studies. Addressing these research gaps and overcoming translational hurdles are critical steps towards fully realising the potential of CB biomaterials in medical applications. Future research efforts should prioritise clinical validation, standardisation of protocols, and compliance with regulatory requirements to facilitate the broader adoption of these innovative biomaterials in clinical practice.

## 5. Future Directions

CB stands poised for avant-garde implementations, capitalising on its unique attributes. Leveraging the advantageous features of CB, such as its porosity, microstructures, and crystallography, has paved the way for the synthesis and enhancement of bone tissue materials. The synthesis of HAp and the innovation of scaffold structures with diverse geometries have ushered in new horizons for medical applications. CB can find utility in the surgical arena as a graft material replacement, offering fresh options as a “smart” biomaterial tailored for craniofacial bone reconstruction and regenerative procedures. Furthermore, injectable HAp synthesis holds promise for treating irregular bone defects. 

Given the compelling mechanical, biological, and physical properties demonstrated by HAp in previous literature, it presents a fitting biological solution for a spectrum of challenges in restorative dentistry. For instance, CB-HAp, enriched with calcium and phosphate ions, emerges as a compelling, biocompatible, and cost-effective alternative for the treatment of demineralised dental tissues.

In the context of endodontic practice, the use of irrigant solutions with bleaching or chelating actions within root canals can inadvertently soften dentinal walls post mineral depletion. Herein lies the hypothesis that incorporating CB-HAp into the final irrigant solution could effectively surmount this challenge. The rationale behind this hypothesis is attributed to the potential ability of CB-HAp to enhance mechanical and physical properties, coupled with its innate outstanding biocompatibility. Moreover, CB-HAp’s potent antimicrobial properties against certain bacterial strains suggest its potential use in treating root canal infections [83,84]. This opens avenues for incorporating this material into root canal sealers, irrigant solutions, intercanal medicaments, implant coatings, and drug delivery systems. CB-derived HAp materials could also increase the microhardness of the internal walls of the roots. Interestingly, since CB-derived materials are rich in calcium ions [4], they could be incorporated into restorative materials; these encompass cement, liners, luting agents, capping materials for dental implants, sealants, and adhesives. However, such hypotheses need to be confirmed through conducting more laboratory and clinical studies. 

Notably, tissue engineering and nanotechnology convergence can yield natural tissue-mimicking designs, culminating in structures akin to dental tissues, including dentin, pulp, periodontal ligament, cementum, and alveolar bone. This confluence holds immense potential for transformative dental science and clinical practice advancements.

For future research, large-scale randomised controlled trials (RCTs) with sufficient sample sizes, robust study designs, and adequate follow-up periods to generate high-quality evidence are crucial. In addition, long-term biocompatibility studies with standardisation for the protocol will enhance result reproducibility, reliability, and comparability across different research studies and clinical trials. Innovative approaches can be applied to improve the use of CB in regenerative medicine, such as by incorporating bioactive molecules or growth factors to promote tissue regeneration and accelerate healing processes [102]. Utilising 3D printing techniques to create patient-specific implants or scaffolds, and investigating the synergistic effects of combining CB with other biomaterials, cells, or therapeutic agents for tailored regenerative treatments, is also promising. Adopting these strategies makes it possible to overcome current limitations, advance scientific understanding, and accelerate the clinical application of CB biomaterials in regenerative medicine and dentistry. These efforts have the potential to pave the way for innovative treatments that improve patient outcomes and quality of life.

## 6. Conclusions

The exploration of CB has revealed its myriad applications across tissue engineering, regenerative medicine, and dentistry. In vitro and in vivo studies have demonstrated CB’s exceptional mechanical, biological, and chemical properties, paving the way for innovative biomedical applications. Its inherent biocompatibility, availability, and cost-effectiveness position CB as a promising therapeutic agent derived from waste materials. This not only underscores the sustainable aspect of utilising byproducts but also signifies a valuable advancement in the field of biomedical science. Additionally, the green synthesis of CB biomaterials highlights their eco-friendly nature, further enhancing their appeal as sustainable and innovative solutions in biomedical applications.

Large-scale RCTs are required to evaluate the efficacy and safety of CB biomaterials in various clinical applications, such as for bone grafts and dental implants. Additionally, extended follow-up studies should be performed to assess CB biomaterials’ long-term biocompatibility and immunogenicity. These suggestions will help improve the utilisation of CB biomaterials in biomedical applications.

## Figures and Tables

**Table 1:** Summarising the key differences and similarities between cuttlefish bone and other marine sources.

Marine Sources	Reference	Porosity	Modulus of Elasticity	Biocompatibility	Strength	Non-Toxicity	Stability Post-Hydrothermal	Biodegradability	Special Components	Biomedical Applications	Environmental Impact
**Cuttlefish Bone**	[25,29,30]	High (~93%)	Optimal	High	High	High	High	Yes	Aragonite, chitin (biodegradable, antimicrobial, hemostatic)	Bone tissue engineering, scaffolds, hydroxyapatite (HAp) synthesis	Reduces biowaste, by transforming raw materials into higher-value products. uses
**Nacre**	[31,32]	Low to moderate	High	High	High	High	low	yes	Aragonite, Organic Matrix	Bone grafts, implants	Generally sustainable
**Coral**	[33,34]	Moderate	Moderate	High	Moderate	High	Low	yes	Calcium Carbonate	Bone grafts, dental implants	Sustainable with some environmental impact due to extraction
**Sea Sponges**	[6,15]	High	Low	High	Low	High	N/A	Yes	Silica and chitin	Tissue engineering scaffolds	Generally sustainable
**Sea Urchins**	[15,31]	High	Low	High	Low	High	N/A	Yes	Magnesium Calcite	Tissue engineering, biomedical devices	Generally sustainable
**Crab Shells**	[4,35]	Low to moderate	Moderate	High	Moderate	High	N/A	Yes	Chitin, Calcium Carbonate	Wound healing, drug delivery, tissue engineering	Generally sustainable
**Oyster Shells**	[4,36]	Low to moderate	Moderate	High	Moderate	High	N/A	Yes	Calcium Carbonate	Bone grafts, dental implants	Generally sustainable

**Table 2:** Studies on CB applications in animal-experiment-based bone regeneration and biomaterial assessment.

Study ID	Aim of the Study	The Methods				Finding/s	Limitations of the Study	Recommendation for Bone Regeneration
		Preparation of CB and Scaffold	Experimental or Commercial Materials	Methods of Characterisation	Preparation of the Animal			
[41]	The aim was to create periosteum substitutes with exceptional osteogenic and angiogenic properties for treating rat calvaria bone defects	1. Preparation cube of the cuttlebone-derived organic matrix (CDOM) by removing calcium carbonate by using hydrochloric acid, phosphoric acid, and ethylenediaminetetraacetic acid and forming CDOM-HCl, CDOM-H_3_PO_4_, and CDOM-EDTA, respectively. 2. Forming “S”-oriented grooves on the surfaces.	Experimental material	1. SEM, EDS, FTIR. 2. Tensile test. 3. Water contact angle. 4. Biocompatibility test on mesenchymal stem cells. 5. Osteogenesis and Angiogenesis Assessment. 6. Micro-CT	1. Male rate of 300–350 g 2. An incision was made in the middle of the skull and stripped out the periosteum. 3. Bilateral defects of 5 mm were created and covered with different CDOM. 4. The suture was closed with the application of antibiotics.	1. CDOM films are highly biocompatible and the S groove is suitable for cell growth. 2. CDOM-EDTA exhibited the highest bone formation neovascularisation and bone regeneration and smaller amount of fibrous tissue (highest osteogenesis). 3. Biosafety of the CDOM films after surgery. 4. No inflammatory change or abnormality in the pathological section (histological safety of CDOM).	1. Mechanical limitations. 2. Eight-week interval.	A highly suitable candidate for clinical use in the regeneration of bone defects.
[42]	To test PVA-based biomimetic CB aerogel scaffolds (ASs) for osteogenesis in the calvarial bone defects of Sprague-Dawley (SD) IGS rats. (in vivo study)	1. The PVA solution one-step rapid freeze-drying method to obtain the PVA-CB AS. 2. PVA/MCNTs formed by adding (modified carbon nanotubes) MCNTs. 3. PVA/MCNT/HAp aerogel scaffolds were suspended in 50 mL of simulated body fluid (SBF) at 37 °C to promote the mineralization of HAp on the scaffolds.	Experimental material	1. TEM, FESEM, RS, TGA, and XRD. 2. Pore size estimation. 3. Compression test. 4. MC3T3-E1, ALP, and gene expression 5. Micro-CT analysis of the rats.	1. Eight-week-old male rats (5 rats in 4 groups). 2. The calvarial defects, 5 mm in diameter, were created on the calvarium using a trephine in each rat.	1. Pore size and MCNTs increased AS hydrophilicity and compression. 2. PVA/MCNT/HAp AS had the best cell adhesion, proliferation, ALP activity, target gene expression, and mineralisation. 3. Micro-CT showed bone development at eight weeks, with PVA/MCNT/HAp-filled bone defects.	Not mentioned.	PVA/MCNT/HAp AS has potential applications for bone regeneration.
[43]	To evaluate the cytotoxicity of the CB xenograft compared to commercial grafts (PerOssal^®^) (in vitro and in vivo study).	Hydrothermal reaction (HT) for preparing HAp from CB by mixing Aragonite and 0.6 M NH_4_H_2_PO_4_ to perform Ca/P; the mixture was heated at 200 °C for 12 h.	Experimental material	1. Biocompatibility (osteoblasts tests) MTT assay for the viability of hMSC. 2. XRD and SEM. 3. Body weight and temperature before and after implantation.	1. Total of 27 rabbits divided into three groups (CB, PerOssal^®^, and control group) 2. Bone graft material was injected into a femoral muscle in two experimental groups (CB + NaCl 0.9%) and Perossal^®^ + NaCl 0.9%), with NaCl solely in the control group.	1. The non-toxic effect on hMSCs for both CB xenograft PerOssal^®^ and pyrogenicity rabbit test was the same for both groups. 2. The properties of HAp from CB are similar to those of human HAp.	1. Only the acute phase (72 h) was measured, not the chronic phase 2. No bioavailability test was used. 3. No figure for XRD was shown.	CB material has potential application as bone graft material.
[44]	To determine the effect of HAp from CB as bone filler on femur bone regeneration in white rats (in vivo study).	Hydrothermal treatment (HT) was used to prepare HAp from CB by adding 1 M CaCO_3_ and 0.6 M NH_4_H_2_PO_4_, then heating the mixture at 200 °C for 12 h, followed by sintering at 900 °C for 1 h.	Experimental material	1. XRD. 2. Light microscope.	1. Thirty rats aged 3–4 months and weighing 200–300 g. 2. CB-HAp and bovine HAp powder (0.5 mg each) were inserted into the fractured femur bone defect (1 mm). 3. Treatment times were 28 and 56 days. 4. Three groups (control, bovine, and CB group).	1. The bone growth and regeneration process were affected by HAp-CB and the recuperation time (56 days). 2. HAp -CB and HAp of bovine bone provided the same result compared to the control group, so they can be used as bone fillers for fracture sites.	Not mentioned.	It can be used for regenerating bone.
[45]	To assess the potential of CB-derived HAp and any potential synergistic effects of platelet-rich plasma with this scaffold on bone healing. (In vivo study.)	CB block was treated with 5% NaClO for 48 h and 0.6 M of NH_4_H_2_PO_4_ to obtain Ca/P 1.67 and the mixture was heated at 200 °C for 24 h. Finally, CB was sterilised with irradiation.	Experimental material	1. XRD. 2. SEM. 3. Fluoroscopy and histopathological examination.	1. Platelet-rich plasma PRP was made from plasma and 8 mL of centrifuged blood. 2. Groups were randomly assigned to use both legs (tibia defect 3.5 mm): negative control, I with PRP, II with raw CB, III with raw CB and PRP, IV with CB/HAp, and V with CB/HAp and PRP. 3. Animals were killed eight weeks after implantation.	1. Raw CB is an acceptable bone defect filler by itself; however, processed HAp is more promising based on histopathologic terms of union and cortical indices. 2. CB is a reliable marine source that promotes the production of new bones.	A more extended evaluation period is needed to accurately evaluate this biomaterial and track the remodelling stage of the bone healing process.	It can be used for bone defect repair.
[46]	1 To evaluate the osteoconductivity of three HAp samples (natural, synthetic, and manufactured) using a sinus lift model in rabbits. (in vivo study). 2. To compare results to biomaterial topography and physical attributes.	1. Bovine hydroxyapatites [BHAp] and CB hydroxyapatite [CBHAp] with high calcined temperature) and synthetic hydroxyapatite (SHAp). 2. In this study, the particle sizes used were 250–1000 µm (BHAp and SHAp) and 500–1000 µm (CBHAp).	Commercial material	1. SEM. 2. BET for specific surface area analysis.	1. Twenty-four mature rabbits (3 kg) with 45-sinus floors. Insulin syringes were used to carefully introduce the substance into the sinus wall and membrane after administering antibiotics and anaesthetic. 2. The sample was separated into enlarged volume, SEM, and newly produced bone. 3. Each group included five samples, and results were assessed after 1, 5, and 12 weeks.	1. With natural HAp, more bone was detected than with synthetic HAp because they have bone-like macro-architectures with interconnected macropores. 2. With BHAp, there was a substantially increased bone-to-material contact, which improved the biomechanical environment.	More characters- action and investigation are needed to identify the biomaterial’s physicochemical properties.	Material–bone contact.
[47]	To demonstrate how the CB affects the speed of healing for long bone fractures.	Injection of the mixture of cuttlebone extract and NaCl 0.9% solution.	Experimental material	1. X-ray photo. 2. Callus index. 3. Histochemical examination.	1. Thirty-two rats were split into control and experimental groups. 2. The right tibia was broken after the incision. 3. Injections of CB and NaCl 0.9% were given to the treatment and control groups, respectively. 4. Leg was repositioned and secured.	CB sped up the healing of fractured bones by raising the synthesis of osteoblasts and forming thicker calluses.	1. Limited period for the study: 2–3 weeks. 2. No characterisation of CB HAp.	Improved bone healing.
[48]	To separate low-molecular-weight sulphated chitosan from CB and extract, purify, and test its anticoagulant (AC), cytotoxic, and antiviral (AV) activities on birds (in vitro study).	1. Researchers blended CB to powder after washing out mud and debris. Chitin was decalcified in a dilute of 1.82% HCl after NaOH and KMnO_4_, and oxalic acid deproteinized it. 2. Sulphated chitosan was obtained by mixing chitosan and chlorosulphonic acid. 3. The substance was dialysed, filtered, freeze-dried, and stored.	Experimental material	1. UV–vis spectroscopy. 2. Fluorescence spectroscopy. 3. FTIR, TGA, and FT-Raman spectra analysis. 4. Elemental analysis 5. DSC thermal analysis. 6. Measurement of the molecular weight. 7. NMR spectroscopy. 8. Anticoagulant assay. 9. Leucocyte migration inhibition assay. 10. Antiviral activity.	RBC button development was monitored during a 30 min incubation period at room temperature with 1% avian RBCs added to the mixture (SP-LMWSC diluted in PBS).	1. CB critical functional groups for SP-LMWSC were extracted. 2. The SP-LMWSC allows long AC sessions and inhibits avian LM. 3. SP-LMWSC prevents avian RBCs from hemagglutinating by binding to ND virus surface receptors. 4. SP-LMWSC’s in vitro AC, cytotoxic, and AV characteristics are promising for further investigations.	Not mentioned.	The material can be used for biomedical purposes and activities.
[49]	To generate a highly porous, nanoscale-sized HAp, a CB at high temperature was employed to improve protein adsorption and bone growth following subcutaneous implant. (In vivo study.)	1. CB blocks were immersed in 5% NaClO for two days to remove protein, then sealed in Teflon-lined st. st. reactors and 0.5 M (NH_4_)_2_HPO_4_ was added. 2. The high-temperature treatment was conducted at 180 °C for 96 h. Samples were collected at 12, 24, 48, and 96 h, then washed in boiling water and dried in an oven	Experimental material	1. SEM. 2. Mercury intrusion porosimetry. 3. XRD and FTIR. 4. Protein adsorption test. 5. MTS, ALP, and OCN tests. 6. Light microscope.	1. Twenty mice aged six weeks were included. Dorsal subcutaneous pockets were created and sterilised with 75% ethanol. A CB block was then implanted. 2. New bone growth was detected using light microscopy. 3. Each specimen had four portions analysed and four pictures selected for each section.	1. CB was utilised to make porous, nanocrystalline HA bone replacements in various shapes. 2. CB/HAp significantly enhances MSCs’ osteogenic phenotype without an osteogenic reagent. 3. High osteoinductive capacity affects protein adsorption and MSC differentiation.	Conducting a quantitative comparison of serum and CB/HAp concentrations is crucial.	It can be used as a bone replacement material.
[50]	To assess the biocompatibility of CB and CB-derived hydroxyapatite (CB/HAp). (In vivo study.)	1. CB1 was formed by defatting and freeze-drying and sterilisation 2. CB2 was obtained by removing organic components, washing, drying, and sterilizing. 3. CB/HAp hydroxyapatite was formed from CB2 using hydrothermal treatment at 200 °C 4. Coral-derived HAp was used to produce CHAp 5. CB block was 5 mm in diameter and 2 mm in thickness.	Experimental material	1. XRD. 2. Histologic investigations. 3. CCD camera-based digital image analysis system.	1. Twenty 9-week-old mice (22 ± 0.2 g) were used for aseptic surgical tech and anaesthesia for the back. 2. Through the incision, sterilised implants were placed subcutaneously with a probable dose of antibiotics.	1. Among the experimental implants, CB/HAp was the most biocompatible material with thinner fibrous capsule thickness. 2. CB2 preparation is more effective than CB1, but there is no difference between the two.	Short time interval: 2–4 weeks.	CB/HAp can be used inside the body.
[51]	To compare CB-implanted rabbit calvarial bone defect healing. (In vivo study.)	1. Defatting and freeze-drying (CB1). 2. Organic component removal (CB2). 3. High temperature at 200 °C/24 h for CBHAp (HAp from CB2). 4. Coral-derived HAp. 5. Hamster ovarian cell expression of CB1 together with rhBMP-2. 6. CB was formed into cylindrical disks with a diameter of 5 mm and a thickness of 2 mm	Experimental material	1. XRD. 2. Radiography. 3. Histological investigation.	1. Twenty-seven 9-month-old rabbits (3.2 ± 0.5 kg). 2. With an aseptic surgical procedure, a sagittal incision was made at the midline of the calvaria after anaesthesia and removal of the skin and periosteum was completed. 3. Antibiotic coverage was provided for three days	1. CBHAp may be more biocompatible for compact bone defect regeneration. 2. Osteoconduction makes CBHA a safe bone graft for compact bone defect models. 3. Group CBHAp had faster bone regeneration at 12 weeks.	Further experiments are needed to identify the characteristics of CB.	CBHAp is a valuable bone graft material with irregular bone defects.
[52]	To assess cuttlefish bone-derived HAp granules (material: CB-HAp) as alternative biomaterials for bone grafts (in vitro and in vivo study).	CB pieces were boiled with 4% NaClO to remove the organic components and HT was at 200 °C with (NH_4_)H_2_PO_4_ for Ca:P 10:6 and the Teflon pressure vessel. The size of CB HA granules ranged from 200 to 500 μm.	Experimental material	1. XRD and SEM. 2. Biological tests. 3. Micro-CT bone analysis.	1. Six adult rabbits (ages > three months) (weighing 2.5–3.0 kg) were anaesthetised and then three separates circular calvaria defects were made. 2. First defect: control; second defect: CB-HAp granules; third defect: pure HAp granules	1. Microporous CB-HAp granules, derived from raw CB, were produced through hydrothermal treatment. 2. Synthetic HAP is less biocompatible than CB-HAp. 3. Pure HAP does not regenerate bone like CB HAp granules. 4. None showed necrosis or foreign body responses.	More research is needed on the mechanical characteristics of CB-HAp granules.	CB-HAp may be used in bone graft substitutes for accelerated bone healing.
[53]	To evaluate the CB xenograft’s capacity for bone regeneration for 24 weeks using radiography and histology (in vivo study).	With the aid of a scalpel blade, the CB was reduced to tiny pieces and disinfected with ethylene oxide.	Commercial material	1. Physiological measurements. 2. Radiographical and biochemical evaluation. 3. Histological examination.	1. One hundred and five one-year-old rabbits were separated into five groups, then seven. 2. CB, demineralised bone matrix, bovine cancellous graft, and TCP filled a 3 mm uni-cortical defect after anaesthesia and periosteum excision (5 mm). 3. Broad-spectrum antibiotics were applied locally	1. Comparing CB to other groups, it was thought to be the best graft that was not rejected and caused no infection. 2. After one week, the fibrous union was initiated with the CB graft. 3. Twenty-eight days after surgery, the bone healing in the CB group progressed more quickly than in the other groups, indicating its effectiveness in orthopedic surgery.	Not mentioned.	CB appears to be a compatible material.
[54]	To synthesise microporous scaffolds by HT by converting CaCO_3_ in CB into calcium phosphate composites (HA and TCP) (in vitro and in vivo).	1. CB were cut into different dimensional cylinders with (NH_4_)_2_HPO_4_ were sealed in a Teflon SS autoclave in the furnace at 180 °C. 3. HT tested between 3 and 48 h in the pH of the solution was 7.8–8.2.	Experimental material	1. XRD, FTIR, and TGA. 2. Mechanical properties. 3. Hemotologial test (APTT), PT, TT. 4. Biological tests.	The prepared scaffold was implanted in the rabbit femur after being stained with eosin.	1. With tunnel-like microstructures, HT can perform CB scaffold for 3 h. 2. MTT showed that macroporous materials were non-toxic and proliferated. 3. Material biocompatibility was determined in vivo and in vitro.	The number of samples and the details of animal preparation were not mentioned	A material’s ability to replace bone was perfected.
[55]	To examine the effect of CB thermal burn (second-degree) injuries in rats and compare them with silver sulfadiazine (SSD) in an in vivo study.	CB was demineralised with HCl, then washed with distilled H_2_O, and precipitated with 4% NaOH for 24 h, followed by washing and drying. The ointment was mixed with white petroleum (Vaseline) with CB in a 6:4 ratio.	Commercial material	1. XRD and FTIR. 2. Microscope and blood test. 3. Lipid peroxidation assay 4. Cytokine measurement	1. Seven-week-old 200–250 g rats were used. After cleaning with ethanol, the exposed area on each rat was heated to 100 °C for 10 min to induce a second-degree burn. 2. The mice were randomly assigned to 12 groups: SSD (0.5 g), CB (0.5 g/cm^2^), white petroleum (0.5 g), and negative control. 3. The ointment was applied twice daily to each group.	1. CB is a potential new material to treat wounds like skin burns as it reduces the inflammation of lipid peroxidation In addition, it promotes re-epithelialisation. Moreover, it accelerates wound healing.	No contribution for XRD.	It can be used for skin injury.
[18]	To create porous HAp from CB that has undergone high temperature and assess its biocompatibility using undecalcified materials (in vivo study).	CB block sinking in 4% NaOCl to remove organic compounds; after washing, 2 M (NH_4_)_2_HPO_4_ was added for 16 h at 180 °C, then immersed again in 2 M (NH_4_)_2_HPO_4_ and treated at 200 °C for 24 h hydrothermally + dried at 90 °C.	Experimental material	XRD, SEM, and TEM.	Rabbits weighing 2.5 kg were prepared with a 5 mm hole diameter made in the femoral condyles, 5–7 mm-long specimens were implanted, and a TEM examined the stained section.	With a good calcium source structure in the CB scaffold, the porous HAp can be used as a biomaterial for bone substitutes as it is biocompatible.	The number of samples was not specified, and the study required additional characteristic tests.	Biocompatibility is to be used as a suitable bone substitute.

Abbreviations: TEM, transmission electron microscope; FESEM, field emission scanning electron microscopy; XRD, X-ray diffraction; TGA, thermal gravimetric analysis; SEM, scanning electron microscopy; FTIR, Fourier-transform infrared.

**Table 3:** Studies on HAp synthesis from CB (HAp group) and its characterisation using various methods.

Study ID	Aim of the Study	The Methods			Finding/s	Limitation
		Preparation of CB	Experimental or Commercial Materials	Characterisation		
[56]	To create a hydrogel composite with HAp-based gelatin and genipin as the crosslinking agent.	1. Researchers washed CB to remove odour and contaminants and then calcine. Phosphoric acid, sodium dodecyl sulphate (SDS), and ammonium hydroxide (AH) were added and dried. 2. Researchers created a gelatin hydrogel composite with HAp, crosslinked with genipin, and dried scaffolds (1 × 1 × 1 cm^3^).	Experimental materials	1. Swelling behaviour. 2. Degradation behaviour. 3. MTT cytotoxicity assay. 4. XRD, FTIR, SEM, TEM, and TGA.	1. The cylindrical and needle-shaped CB-HAp can be used as a filler when a hydrogel is formed using gelatin as the matrix. 2. Hydrogels with less than 1% HAp are biocompatible with low SDS and are ideal scaffolding materials.	Not mentioned.
[57]	To create HAp, nano-powders using CB, mussel shells (MSs), chicken eggshells (ESs), and synthetic bioinspired amorphous calcium carbonate ACC.	1. ESs, CBs, and MSs were washed, boiled, dried, ground, and sieved to remove particles larger than 300 µm 2. HAp of ESs, CBs, MSs, and ACC via wet mechano-synthesis with +(NH_4_)_2_HPO_4_ or H_3_PO_4_ was used to obtain CaP:1.67, then ball-milling and oven drying were performed for 24 h. 3. HAP uniaxial pressing and sintering produced ACC-800, ES-900, MS-1000, sHA-1100, CB-900, and CB-1100.	Commercial materials	1. XRD. 2. ICP/OES. 3. SEM. 4. Cytotoxicity test. 5. LDH cytotoxic assay. 6. Confocal analyses for cell adhesion.	1. Bioactive Ca/P nanomaterials can be produced by synthesising nanocrystalline HAp from CBs, ESs, MSs, and ACC and consolidating them between 800 and 1100 °C. 2. Mg^2+^ in ES-derived HA and Sr^2+^ in CB-derived HAp affect the crystalline phases in addition to Ca/P. 3. Materials produced with good cell adhesion qualities and no cytotoxic effect are appropriate for bone regeneration.	Not mentioned.
[58]	To prepare HAp nanocomposites (HAp-NC) using an oil bath-mediated synthesis method and study their mechanical and biological properties.	1. The CB lamellar part was cleaned with water, acetone, and ethanol to remove impurities and then dried in a hot air oven. The powder was milled with a high-energy ball mill and then 0.6 M of (NH_4_)_2_HPO_4_, pH 8 to 12, was added and mixed, washed, dried, and sintered. 2. Graphene oxide (GO), carbon nanotubes (CNTs), multi-walled carbon nanotubes (GONRs), and silver nanoparticle (Ag NP) nanocomposites were added with different concentrations of 1, 3, and 5 wt%.	Experimental material	1. XDR, FTIR, and SEM. 2. MTT assay. 3. Hemolysis. 4. Antimicrobial activity. 5. Vicker’s hardness test. 6. Bioactivity test. 7. Drug loading and release study.	1. Oil-path-synthesised HAp-NC with rod-like morphology, carbon, and Ag NPs increased crystallite and particle size but decreased hardness due to agglomeration above 5 wt% carbon. 2. Ag NP-containing nanocomposites have higher inhibition for *E. coli* and *S. aureus.* 3. The matrix’s sheet-like structure controls lidocaine release. 4. Biocompatibility may make this nanocomposite suitable for load-bearing biomedical applications.	Not mentioned.
[59]	To synthesise an injectable bone-active hydrogel containing hyaluronic acid (HA) and silk FIB to mimic the extracellular matrix (ECM).	1. Bombyx Mori cocoon dissolving yielded silk fibroin (FIB) solution. 2. CB-treated HT was heated to remove organic materials. Then, 0.6 M NH_4_H_2_PO_4_ was sealed in a Teflon lining, heated in a furnace, washed, and ground into a powder 3. CB HAp and hyaluronic solution were stirred, then sonicated, centrifuged, and oven-dried. 4. FIB-HA-HAp crosslinking was performed.	Experimental material	1. FTIR, XRD, TGA, and SEM. 2. DLS measured the size and Zeta-Sizer. 3. Viscosity analysed. 4. Biological and antibacterial tests.	1. Silk fibroin HA-HAp hydrogel is biocompatible and natural. 2. Porous, interconnected, and viscous HA-HAp-FIB cell communication promotes osteoblast attachment without cell toxicity. 3. HA-HA-FIB hydrogel outperforms other hydrogels in mechanical strength. 4. At 15 μg/mL concentration, *S. aureus* is inhibited more effectively than *E. coli*.	Not mentioned.
[60]	To investigate ball-milling biogenic CaCO_3_ sources such as mussel shells (MS), eggshells (ES), and CB in aqueous circumstances at low temperatures to produce HAp.	Researchers washed, boiled, dried, ground, and sifted the following ingredients using a centrifugal mill: (1) (NH_4_)_2_HPO_4_, with an initial pH of 8.5; (2) ammonium phosphate dibasic and hydroxide, with an initial pH of 13; and (3) H_3_PO_4_, with a starting pH of 3.2. After aqueous milling, the slurry was dried at different temperatures.	Experimental materials	1. XRD. 2. FTIR. 3. FE_SEM. 4. TGA. 5. ICP/OES.	1. Bone-like carbonate apatite synthesis was achieved. 2. CB bone aragonite rapidly converted to HAp in acidic aqueous environments. 2. The flake-like crystals were nanometric. 3. Powder may have the potential to be bone tissue engineering biomaterials.	Not mentioned.
[32]	To use Arrhenius kinetics to study how aragonite from CB is transformed into HAp based on temperature and reaction time.	1. To eliminate organic compounds from CB, heat at 350 °C for three hours and add 0.6 M NH_4_H_2_PO_4_ solution to achieve Ca/P = 1.67. 2. The teflon-lined stainless steel pressure vessel was heated from 140 to 220 °C for 20–48 h in an electric furnace, rinsed, and dried at 110 °C.	Experimental material	1. SEM. 2. XRD. 3. FTIR. 4. Crystallisation kinetics	1. Aragonite entirely became HAp over time. 2. The hydrothermal treatment temperature preserved aragonite’s interconnecting porous architecture, enhancing HA production. 3. One-dimensional development governed by diffusion is the best way to forecast HA crystallisation and the morphology gives a 3D structure for bone tissue creation.	Not mentioned.
[61]	To study the hydrothermal conversion of CB aragonite to HAp at 200 °C for 1–48 h.	1. CB pieces were heated to 350 °C for 3 h to eliminate organic matter, then 0.6 M of NH_4_H_2_PO_4_ solution was added to set Ca/P = 1.67. A 200 °C Teflon-lined stainless steel pressure vessel was employed. 2. Researchers washed and dried the HAP at 110 °C.	Experimental material	1. XDR, FTIR, SEM. 2. DSC-TGA. 3. Hg intrusion porosimeter to detect CB porosity.	1 Complete conversion of CB aragonite to HAp was achieved in 48 h with NH_4_H_2_PO_4_ at 200 °C. 2. Hydrothermal treatment converts aragonite to HAp while preserving interconnecting channels and maintaining a plate- and needle-like crystal morphology.	Not mentioned.

Abbreviations: ICP/OES, Inductively Coupled Plasma Optical Emission spectroscopy; TEM, transmission electron microscope; FESEM, field emission scanning electron microscopy; XRD, X-ray diffraction; TGA, thermal gravimetric analysis; SEM, scanning electron microscopy; FTIR, Fourier-transform infrared.

**Table 4:** Preparation methods, characterisation, and key findings of CB-derived scaffolds (scaffold group) for BTE.

Study ID	Aim of the Study	The Methods			Finding/s	Limitation
		Preparation of CB	Experimental or Commercial Materials	Characterisation		
[62]	To use 3D printing to create scaffolds from eggshell ES, pearl, turtle shell TS, degelatinised deer antler DDA, and CB, five biominerals with L-polylactic acid (PLLA).	1. Researchers washed, dried, ground, and filtered ES, pearl, TS, DDA, and CB. 2. PLLA and powder were dissolved and dispersed and ink was added to the 3D printer barrel. 3. Researchers put the scaffolds in the freeze dryer to form them. 4. Pure PLLA was set as control.	Experimental material	1. FTIR, XRD, PSD. 2. Viscosity test. 3. SEM after gold spray. 4. Cell live/dead staining. 5. Cell proliferation assay. 6. ALP detection.	Three-dimensional porous scaffolds (SCs) can be created from organic materials, such as 3D printing ink with PLLA. The five types of SCs used by the researchers included both macroscopic and microscopic pores, which facilitated cell migration, proliferation, and adhesion, and were biocompatible for bone tissue engineering (BTE).	No mechanical test.
[63]	To create paste-like 3D printing inks with an extracellular matrix (ECM)-inspired formula based on marine ingredients including sodium alginate (SA), CB, and fish gelatin (FG).	1. Researchers washed, dried, and ground CB block. 2. Comparison of FG, SA, and CB powder ink formation to controls was used. Mixing was essential for ink uniformity. 3. Bio CAD software (Developed by, Switzerland) and post-printing crosslinking were utilized for the design of 3D scaffolds (3D-SC).	Commercial materials	1. Mechanical tests. 2. Structural and dimensional tests. 3. Morpho- and microstructural test. 4. In vitro biocompatibility tests.	1. Marine resources resembled ECM. Calcium-rich macro-porous square pores were present in 3D SC. 2. Alginate and crosslinking stabilised composite SC. 3. Biocompatibility testing showed that all composites were bone-implantation scaffolds. 4. Thick-paste inks for scaffolds used in hard tissue regeneration and repair were observed	Restrictions for 3D-printable hydrogel precursor and low shape fidelity and resolution.
[64]	To create scaffolds (SCs) with uniform or variable porosity as defined by P-3DP. CB nano-CaP powder was utilised.	1. Researchers centrifugally ground, washed, boiled, and dried CB. 2. Ball-milling H_3_PO_4_ and Ca/P = 1.67, drying, heating, and adding glass–ceramic material generated CaP. 3. Scaffolds were disk-shaped in CAD models.	Commercial materials	1. PSD, TGA/DTA, XRD, FTIR, SEM, and ICP were used. 2. SC characterisation. 3. Biological evaluations.	1. Cell proliferation was similar in two SC nano-BCP geometries produced from CB combined with glass–ceramic powder. These nanoparticles increased SC porosity. 2. Gradient pores and CB-derived powder reduced metabolic activity, but adhesion of hMSCs was achieved quickly	No mechanical test.
[65]	To demonstrate how bioinspired 3D printing can generate high-performance cellular materials.	1. Dried CB samples, designed with three different models, all featured the same lamellae structure but varied in wall design: 1. Straight wall. 2. Symmetric S-shaped model. 3. Asymmetric distorted S-shaped wall.	Experimental materials	1. SEM, TGA, and micro-CT. 2. Finite element method (FEM).	CB-like cellular material, derived from 3D printing, is a bioinspired material with lightweight properties, high strength, excellent energy absorption, and wavy walls, making it suitable for engineering applications and implantable devices.	Not mentioned.
[66]	A novel organic/inorganic paste-type ink with cuttlefish bone was developed to manufacture 3D-printed scaffolds (SCs) for bone abnormalities.	1. CB lamellar components were washed, dried, crushed, and ground into powder. 2. Fish gelatin (FG), CB, and sodium alginate (SA) were mixed to make paste-type ink, and SC was 3D bioprinted, crosslinked by glutaraldehyde (GA) for 48 h, cleaned, and dried.	Commercial materials	1. Gel fraction (GF) analysis. 2. Micro-CT. 3. Swelling behaviour evaluation 4. Dimensional stability. 5. FTIR. 6. Mechanical test.	1. Concentrated ink improves print quality. 2. Effective crosslinking predicts hybrid SC stability. 3. Swelling, structural changes, and degradation testing showed scaffold stability in PBS, making it an appealing bone replacement for bone tissue regeneration.	Not mentioned.
[67]	To determine how Mg^2+^ ion concentration affects CB-derived calcium phosphate mechanical, biological, and microstructural properties.	1. CB lamellae were treated with NaClO for 48 h, washed, and mixed with NH_4_H_2_PO_4_ and MgCl_2_ in a teflon-lined stainless steel at 200 °C for 48 h. 2. Five samples and a (Ca + Mg)/P molar ratio of 1.67 were prepared.	Experimental material	1. XRD, FTIR, TGA, SEM, and EDAX. 2. Biocompatibility test. 3. Immuno-histochemical test. 4. Isolation of total RNA and PCR (RT-qPCR) analysis. 5. Mechanical test.	1. HT made highly porous bone-mimetic with varied HAP: WH ratios. 2. Magnesium perchlorate was a good Mg^2+^ supply for biphasic SC’s HAP: WH phase ratio, which had better CS and osteogenic differentiation than pure HAP scaffold. 3. Mg-substituted scaffolds are non-cytotoxic and ideal for bone tissue engineering.	Not mentioned.
[68]	Regenerated cellulose and CB microparticles were used to create 3D scaffolds using a non-hydrolytic sol-gel method.	Researchers ground CB in an agate mortar, sifted and washed it at 80 °C with 0.5 M NaOH and 1% SDS (SO_4_ ion source), and dried it. 2. SC became porous by immobilising CB microparticles in RC gel and lyophilisation. 3. RC/CB, RC/CB-SBF, and RC scaffold control sols were employed.	Commercial materials	1. XRF, SEM/EDX, FTIR, and TGA. 3. In vitro cell culture, cytotoxicity, and biocompatibility tests.	1. CB reinforcement and bio-inspired coating impacts on 3D porous cellulose-based scaffold osteoconductiveness. 2. Non-toxic CB extracts did not affect cell proliferation; CB-SC coating in 10× SBF improved biomineralisation. 3. To maximise BTE action, mix CB with polymer gel and coat the scaffold surface with 10× SBF.	No mechanical test.
[69]	To synthesise the highly porous PCL-coated HA made from CB subjected to physicochemical analysis.	1. CB pieces were treated with NaClO, sealed with 0.6 mol NH_4_H_2_PO_4_ and Ca/P 1.67 in a teflon-lined stainless steel, rinsed, and dried at 105 °C. 2. The study involved using a 10% PCL + PLA mixture, where polymer pellets were vigorously stirred in chloroform with varying volume ratios. This mixture was then vacuum-impregnated into HA scaffolds and allowed to dry.	Experimental material	1.FTIR, XRD, SEM and TGA. 2. Mechanical test. 3. Bioactivity test. 4. Cytotoxicity test.	1. The load-bearing properties of the HA/PCL/PLA scaffold can be customized by combining PCL with various types of PLA to adjust brittleness and degradation behavior. 2. HA/PCL/PLA scaffolds are bioactive and non-cytotoxic, stimulate cell adhesion and proliferation due to calcium phosphate deposition, and can be used to develop artificial grafts.	The dynamic culture of osteogenic or stem cells is required to validate potential.
[70]	To make an osteoconductive cellulose-based composite SC with cuttlebone-derived hydroxyapatite (CB-HAp) filler for bone tissue engineering and investigate coastal CB specimens’ elemental compositions.	1. CB was crushed, milled, and sieved (80 µm); HT with NH_4_H_2_PO_4_ and Ca/P molar ratio of 1.67 were used, keeping the suspension inside a 50 mL PTFE (polytetrafluoroethylene) vessel at 200 °C. 2. CB-HAp powder was combined with cellulose gel for 30 min and freeze-dried to make scaffolds porous. 3. Two groups were used: RC and CB-HAp. The control scaffolds were made of regenerated cellulose (RC).	Commercial materials	1. FTIR (KBr), XRD SE, and XRF. 2. Micro-computed tomography. 3. Mechanical test. 4. In vitro cytotoxicity test included: i. Cell proliferation. ii. ALP and OCN activity. iii. Biomineralisation.	1. An osteoconductive porous scaffold from regenerated cellulose and CB-HAP improved SC’s mechanical characteristics using CB microparticles. 2. Trace elements activate biological activity and HAp helps PCL scaffold nucleate minerals. 3. The SC is biocompatible for BTE.	Not mentioned.
[71]	HT CB-derived BCP scaffolds were doped with Sr^2+,^ Mg^2+^, and Zn^2+^ in various combinations and coated with polymeric materials (PCL, PDLA, PEA, and PEU) to create SC.	1. CB was cut, washed, dried, and mixed with (NH4)2HPO4 to obtain non-doped BCP-SC. Meanwhile, cationic nitrate precursor was added to create doped scaffolds. All mixtures were sealed in PTFE at 200 °C for hydrothermal treatment, then washed and dried. 2. BCP scaffolds were coated with PCL, PDLA, PEA, or PEU under partial vacuum for 5 min and then dried.	Experimental material	1. XRD, TGA, ICP SEM and FTIR. 2. Micro-computed tomography. 3. In vitro biomineralisation.	1. CB was converted entirely into a BCP scaffold for undoped and doped SC without altering porosity. 2. Mg^2+^ or Zn^2+^ increases the strength of β-TCP peaks. 3. PEU promotes CS registration for human trabecular bone better. 4. Scaffolds showed potent in vitro SBF bioactivity, making them suitable for bone tissue engineering.	Not mentioned.
[72]	To create 3D scaffolds from mechanically immobilised CB microparticles and regenerated cellulose using a non-hydrolytic sol-gel method.	1. CB were crushed, sieved, rinsed, stirred, and heated with NaOH+ SDS to deproteinize them, then washed and dried. 2. CB immobilised by RC gel, HAP filler, and lyophilisation created a porous structure. 3. RC/CB, RC/CB-SBF (10× SBF), and RC scaffold control.	Commercial materials	1. XRD, SEM, FTIR TGA and EDAX. 2. In vitro cell culture tests: cytotoxicity, cell attachment, ALP and OC tests, and biomineralization.	1. The scaffolds included 9% dorsal chitin, 5% lamella, and were coated with 10× simulated body fluid (SBF) to promote biomineralization. All scaffolds were non-cytotoxic and stimulated cell attachment through β-chitin 2. To achieve osteoconductive characteristics in cellulose-based scaffolds, mix CB with polymer gel and coat the scaffold with 10× SBF. These scaffolds are good candidates for BTE	Not mentioned.
[73]	To explore the possibility of using unprocessed, surface-modified CB to replace pieces of bone.	1. CB pieces were washed, dried, sanitised, and submerged in fresh SBF to produce CB-HA scaffolds. 2. CB-HA-COL scaffolds with 1 mL collagen type 1 were used. 3.Homogeneous collagen was air-sprayed onto the surface, coated, and then freeze-dried for 24 h	Experimental material	1. SEM and EDS. 2. Mercury porosimetry. 3. Compressive strength. 4. Biocompatibility tests.	1. Unprocessed CB with surface modification potential can be employed as a porous, vital bone substitute. 2. Bone marrow mesenchymal stem cell (BMMSC) proliferation and alkaline phosphatase activity were increased) in vitro. The scaffold can be used for bone tissue engineering	Not mentioned.
[74]	To improve and contrast the compressive strengths of PCL and PVA-coated SC made from CB.	1. After cleaning, washing, and drying CB, NH_4_H_2_PO_4_ was heated and added in a Teflon jar at 200 °C with Ca/P = 1.67, then washed and dried. 2. The SC was dipped into chloroform-dissolved PCL pellets under vacuum for total infiltration, and the same was repeated for PVA using de-ionised water.	Experimental material	1. Simulated body fluid (SBF), SEM, FTIR, XRD. 2. Compressive strength. 3. Porosity (P) measurement 4. In vitro biocompatibility tests.	1. Creating HAp from CB while preserving its porous, well-connected, parallel-pillar structure 2. Coated SC retains mechanical integrity while non-coated SC disintegrates in solution due to an apatite layer. 3. PVA and PCL increased CB-HA SC compressive strength without cytotoxicity.	Bone differentiation assay is needed to evaluate the potential role of these materials in BTE.
[75]	Several ratios of CB and sol-gel additives (P_2_O_5_, Na_2_CO_3_, KH_2_PO_4,_ and CaO) were added to both micro- and nanoscale HA powders to create novel bone grafts.	1. CB was washed, dried, and grounded, mixed with HA and additives (P_2_O_5_, Na_2_CO_3_, KH_2_PO_4_, and CaO). The mixture was processed with an ultrasonic homogenizer, dried, and sintered in a vacuum. 2. The prepared specimens had a 10 mm diameter and a 1 mm height.	Experimental material	1. XRD, FTIR, SEM. 2. Hardness test (Vickers test).	1. Bone grafts can be replaced with CB-HAp and B-TCP. 2. Higher additive weights result in larger grains and a more crystalline structure 3. These bio grafts could be used for orthopaedic hard tissue applications, loading human mesenchymal stem cells, or bone regeneration. 4. The highest microscale and elongation HVs were M-H30S40 and N-H30S30 while the lowest were M-H30S20 and N-H30S40. 5. N-H30S40 has the highest nanoscale HV while N-H30S20 has the lowest.	Needed biological or bioactive test.
[76]	To prepare PCL-coated highly porous HAp scaffolds made of CB with straightforward and affordable techniques.	1. Small pieces of CB were treated with (NaClO) and then sealed with 0.6 M NH_4_H_2_PO_4_ Ca/P = 1.67 in Teflon with a pressure vessel at 200 °C, then the SC was washed and dried. 2. Researchers impregnated HAP scaffolds with PCL solution in chloroform using the vacuum impregnation unit VIU.	Experimental material	1. In vitro bioactivity test. 2. XRD. 3. FTIR. 4. SEM. 5. EDX. 6. Compression tests.	1. HT at 200 °C transformed natural aragonite from CB to HAp, preserving its uniquely linked porosity structure and PCL coating. 2. Pure and HAp/PCL-SC include calcium phosphate (CP), generating bone-like apatite. 3. PCL coating on HAp greatly improved scaffold mechanical properties. 4. Since it enhances cell adhesion, proliferation, and differentiation, the SC may help BTE.	Not mentioned.
[77]	To study scaffold microstructure, chemical analysis, porosity, and compressive strength concerning composite mass ratio.	1. CB fragments were mixed in NaOH, crushed, and sterilised by gamma radiation. 2. Washing off soluble salt particles produced porous cylindrical rods of PCL, CB, and sodium chloride. The freeze dryer finished it.	Experimental material	1. SEM and inductively coupled plasma mass spectrometry (ICP-MS). 2. Mechanical and chemical tests. 3. Porosity measurement.	1. Salt particle leaching formed porous PCL/CB scaffolds. 2. By adjusting salt particle size and salt amount, pore size and porosity may be controlled, making SC microporous ideal for tissue engineering. 3. Increased CB and salt chloride consumption affected scaffold morphologies dramatically. 4. Porosity decreases CS of PCL/CB scaffolds, although sodium chloride salt concentration increases porosity.	Not mentioned.
[78]	To exhibit the biocompatibility and osteoblast development of hMSCs grown on natural cuttlefish bone.	1. CB was washed and dried, then cut into blocks of 4 × 4 × 1 mm^3^ and 10 × 10 × 2 mm^3^. 2. Each block was sterilised with Ethylene Oxide (EO) gas for tissue culture dishes.	Experimental material	1. Mesenchymal stem cell culture of hMSCs. 2. Cell viability and cytotoxicity. 3. Osteoblast differentiation of hMSCs. 4. SEM and gene expression. 5. ALP activity.	1. The lamellar region provides more profound cell development for hMSCs than the dorsal part. 2. There is no cytotoxicity with CB heavy metals. 3. Natural CB promotes hMSC adhesion, proliferation, and differentiation. 4. Even though the lamellar component is fragile, CB parts can limit fibroblast infiltration around the bone defect during bone repair.	Further study for mechanical strength and stability.
[79]	To explore CB ways to produce a porous HAp scaffold utilising HT at low temperatures, KH_2_PO_4_, and a quick method.	1. To remove organic material, the CB block was put in NaClO overnight, then washed and dried. 2. KH_2_PO_4_ of 0.16 M to obtain Ca/P = 10/6 with HT was performed at 200 °C for 4 h, followed by washing and drying.	Experimental material	1. XRD, FTIR, SEM and TGA. 2. Biological tests.	1. HT converted CB entirely into biomimetic HAp scaffolds in a short time, maintaining the CB’s original architecture. 2. Good biocompatibility with biological testing was noted.	Dynamic cell culture should be further considered.
[20]	The goal was to preserve the natural CB microstructure while converting it to porous BCP scaffolds with varying HAp and β-TCP compositions.	1. After washing and drying, CB cubes were vacuum-immersed in phosphoric acid and dried in air. 2. CaCO_3_ was decomposed to CaO at 800 °C for 2 h, and then heated to either 1200 °C or 1300 °C for 1 or 3 h.	Experimental material	1. XRD, SEM, TGA, differential scanning calorimetry, and energy-dispersive spectroscopy. 2. Compressive strength (CS).	1. CB converted to porous BCP with varied HA and B-TCP compositions and retained >90% porosity. 2. The load was preserved when various lamellae/pillar configurations collapsed without catastrophically failing BCP-SC under compression. 3. CB porous BCP scaffolds are good bone substitute alternatives for research.	No biological test.
[21]	CB was showing promise as a low-cost supply of materials with a biological origin that could hydrothermally (HT) be converted into HA scaffolds.	Heat-treated fresh CB was converted powder and mixed with NH_4_H_2_PO_4_ to achieve a Ca/P ratio of 1.67. The mixture was placed in a teflon-lined stainless steel autoclave, which was then put in a furnace for hydrothermal treatment at 200 °C.	Experimental material	1. DTA/TG, XRD, FTIR/KBr pellets, FE-SEM and EDS. 2. Biocompatibility MTT assay and ALP/BCIP–NBT assay.	1. HT completely transfers CB into AB-type carbonated hydroxyapatite, similar to human bones. 2. Channelled structure aids solution dispersion and fast kinetic biological activity like BTE. 3. Cheap, readily available natural materials are biocompatible.	No biological test.
[37]	To create a novel method by hydrothermally transforming CB to produce highly bioactive bone scaffolds of HAp	CB pieces were mixed with NH4H2PO4 to set Ca/P = 10/6. Then, the materials were put in teflon-lined stainless. Autoclaves were placed in a furnace at 200 °C, followed by dryness and sintering between 1000 °C and 1400 °C.	Experimental material	1.XRD, FTIR, and FESEM. 2. Biological test. 3. Osteoblasts’ viability and Alkaline phosphatase production.	1. CB aragonite was converted to HAp at 200 °C through a β-TCP transition, which ensured thermal stability 2. CB is cheap, available, and utterly customisable while preserving a porous, linked structure. 3. Bioactivity in SBF and osteoblast biocompatibility make CB-SC appropriate for BTE and implants.	

Abbreviations: ICP/OES, Inductively Coupled Plasma Optical Emission spectroscopy; TEM, transmission electron microscope; FESEM, field emission scanning electron microscopy; XRD, X-ray diffraction; TGA, thermal gravimetric analysis; SEM, scanning electron microscopy; FTIR, Fourier-transform infrared.

**Table 5:** Effects of CB-derived HAp on glass ionomer cement (GIC group) mechanical properties.

Study ID	Aim of the Study	The Methods			Finding/s	Limitation
		Preparation of CB	Experimental or Commercial Material	Characterisation		
[80]	To examine the compressive strength (CS), breaking strength (BS), and compressive modulus (CM) of standard GIC (Fuji IX) modified with 3% TiO_2_ NP(nano-particles), 3% md-HAp, and a mixture of 1.5% TiO_2_ + 1.5% md-HAp.	1. CB synthesis of HAp at 45 µm 2. GIC+ HAp, TiO_2_, and fluoro-aluminosilicate glass powder were uniformly mixed: 3:6 powder/liquid. 3. Particle-free control group and three experimental groups: 3 and 1.5 wt% TiO_2_ and HAp.	Experimental material	1. FTIR. 2. SEM. 3. CS, BS, and CM testing; cylinder-shaped samples were made for each group.	1. Due to the low p/l ratio, GIC powder with TiO_2_ NP and microparticles of md-HAp did not increase CS, CM, and BS because there were too many unreacted particles. 2. TiO_2_ and md-HAp considerably lowered the mechanical characteristics of Fuji IX CS, BS, and CM, but TiO_2_ NP and HAp did not improve them.	1. Hand mixing was used as it was not easy to mix the cement. 2. Limited characterisation of HAp only by FTIR.
[81]	To assess CB-HAp synthesised by hydrothermal (HT) treatment and its effect on conventional cure and resin-modified glass ionomer cement’s microhardness (MH), surface roughness (SR), and fluoride release (FR).	1. Researchers created highly porous CB-HAp with <180 µm via HT. 2. Fuji II and Fuji IX were mixed with HAp powder (2, 5, and 10 wt%) to make homogenous powder (three experimental groups) and a fourth group without HAp. 3. The sample was tested for microhardness, FR, and SR in Teflon moulds.	Experimental material	1. MH and SR testing. 2. SEM. 3. Fluoride measurements by ion-selective electrode.	1. Adding CB micro-HAp to Fuji IX and Fuji II powders does not boost SR. 2. All groups except Fuji II (10 wt%) HAp demonstrated microhardness decreases after adding HAp. 3. Fuji IX samples with (10 wt%) HAp had good FR while (2 and 5 wt%) enhanced FR at all three time periods.	1. Mastication pressures, heat change, and saliva exposure were investigated differently for each sample. 2. No extensive range of GIC was tested. 3. While the findings were clinically relevant, they were not clear, which represents a limitation
[82]	To assess how the mechanical properties of glass ionomer cement (GIC) Fuji II and Fuji IX were affected by the addition of HAp made from CB.	1. HAp from CB prepared by HT method and <180 µm in size were used. 2. Fluoro-alumino-silicate glass was mixed manually with HAp for homogenous distribution. 3. Three groups were modified by incorporating 2, 5, and 10 wt% HAp, and the fourth was without HAp. 4. A split silicone mould was prepared with a polyester strip to prevent air trapping.	Experimental material	1. Compressive strength (CS). 2. Flexural strength (FS). 3. Diametral tensile strength (DTS).	This study found that adding micro-HAp particles derived from CB to Fuji IX and Fuji II powders did not improve chemically set Fuji IX groups, CS, DTS, or FS. Still, it did improve Fuji II groups’ mechanical properties, and 10% HAp significantly improved FS.	The manual spatulation resulted in air inclusions and the inability to obtain the manufacturer-recommended liquid-to-powder ratio.

Abbreviations—FESEM: field emission scanning electron microscopy; XRD: X-ray diffraction; SEM: scanning electron microscopy; FTIR: Fourier-transform infrared.

**Table 6:** Preparation, material synthesis, characterisation, and applications of CB-derived materials in scientific studies (other).

Study ID	Aim of the Study	The Methods				Finding/s	Limitation
		Preparation of CB	Experimental or Commercial Material	Preparation of Materials	Characterisation		
[83]	Testing powdered cuttlebone’s antibacterial activity against *Aspergillus flavus*, *Klebsiella oxytoca*, and *Staphylococcus aureus.*	Electric milling formed CB powder, which was then sterilised before being added to Ringer’s solution.	Experimental material	1. Researchers inoculated bacterial and fungal species by sterile swab in sterilised agar. 2. Negative and positive control groups were used.	1. FTIR 2. FESEM and 3. EDS-mapping 4. Agar well diffusion technique	1. Affordable material with antibacterial against *K. oxytoca* rather than *S. aureus* 2. High inhibition zone was found against *A. flavus.* 3. CB powder has better antibiotic efficiency than synthetic ones.	Not mentioned
[84]	1. Synthesis of nano-rod HAp by oil-bath precipitation method. 2. In vitro antimicrobial tests. 3. Human blood haemolysis.	1. CB powder was mixed with (NH_4_)_2_HPO_4_ and NH_3_ to adjust pH 8–12 with an oil bath to maintain the reaction temperature. 2. A top-down approach using high-energy ball milling was utilized.	Experimental material	1. Fresh bacterial cultures were used. 2. The CB-HAp nanoparticle (NR) samples were poured into nutrient agar.	XRD, FTIR, TEM TGA, antibacterial test and hemolysis test	1. Greater bactericidal activity against *S. aureus* compared to *E. coli* and blood cell biocompatibility were observed 2. HAp NRs can be employed as hard tissue implants because they mimic human bone.	Not mentioned
[85]	To make cuttlefish bone antacid tablets and evaluate the antacid effects of those tablets (in vitro study).	To remove CB odour, the powder was cleaned and dried before being powdered and well mixed to a 60–100 mesh size, then completely calcined.	Experimental material	The direct compression method employed crude CB powder, cellulose, lactose, and starch to make CB: filler tablets at 50:50, 60:40, 80:20, and 90:10 ratios. 2. Researchers compared prepared samples to marketing tablets.	1. CHN, XRF, XRD and FTIR. 2. Flowability. 3. Friability. 4. Weight uniformity, hardness and disintegration test 5. Antacid capacity.	1. The 90/10 and 80/20 ratios were physicochemically and antacidally effective. 2. It is a safe, effective, and economical natural marine antacid with fewer side effects than commercial tablets.	Not mentioned
[86]	To create a composite material with ZnO by sourcing chitin as β-chitin from *Sepia officinalis*.	1. The CB was washed thrice and then treated with CH_3_COOH for demineralisation and NaOH to remove protein. 2. Colourless scaffolds were sealed and kept in plastic containers.	Experimental material	1. A transparent Zn(CH_3_COO)_2_ solution was introduced to a pH 14 chitin membrane after adding NaOH. 2. ZnO-covered chitin template was used. 3. ZnO particles comprised the chitin template-free control group.	1. Stereo and laser confocal microscopy CM. 2. SEM, TEM EDX and RS. 3. Zeta potential. Antibacterial activity	1. B-chitin membrane is utilised as a thermostable organic template for HT development of ZnO nanocrystals to produce chitin/ZnO materials. 2. Antibacterial activity against Gram-positive rather than Gram-negative bacteria is present in chitin/ZnO composites.	Not mentioned

Abbreviations—FESEM: field emission scanning electron microscopy; XRD: X-ray diffraction; SEM: scanning electron microscopy; FTIR: Fourier-transform infrared; CHN: carbon, hydrogen, and nitrogen elements; XRF: X-ray fluorescence.

## Data Availability

Data will be made available on request.
